# Extracellular Chromatin Triggers Release of Soluble CEACAM8 Upon Activation of Neutrophils

**DOI:** 10.3389/fimmu.2019.01346

**Published:** 2019-06-14

**Authors:** Matthieu Ribon, Julie Mussard, Luca Semerano, Bernhard B. Singer, Patrice Decker

**Affiliations:** ^1^Li2P, University of Paris 13, Sorbonne Paris Cité, Bobigny, France; ^2^Inserm UMR 1125, Li2P, Bobigny, France; ^3^Rheumatology Department, Avicenne Hospital, AP-HP, Bobigny, France; ^4^Institute of Anatomy, University Hospital, University Duisburg-Essen, Essen, Germany

**Keywords:** extracellular chromatin, inflammation, neutrophils, autoimmune diseases, soluble CEACAM8 (soluble CD66b), immunomodulation

## Abstract

Increased concentrations of extracellular chromatin are observed in cancer, sepsis, and inflammatory autoimmune diseases like systemic lupus erythematosus (SLE) or rheumatoid arthritis (RA). In SLE and RA, extracellular chromatin may behave as a danger-associated molecular pattern (DAMP). Polymorphonuclear neutrophils (PMN) are described as typical pro-inflammatory cells but possess also immunoregulatory properties. They are activated in SLE and RA but surprisingly remain moderately studied in these diseases, and especially the disease-associated stimuli triggering PMN activation are still not completely characterized. PMN express plasma membrane carcinoembryonic antigen-related cell adhesion molecule (CEACAM) 8 (CD66b) and secrete a soluble form of CEACAM8 after activation. Soluble CEACAM8 has in turn immunoregulatory functions. However, few natural stimuli inducing soluble CEACAM8 secretion by PMN have been identified. Here we demonstrate for the first time that extracellular chromatin triggers secretion of soluble CEACAM8 by primary human PMN. Priming of PMN was not required. Secretion was associated with activation of PMN. Similar induction of soluble CEACAM8 release was observed with purified mono-nucleosomes as well as long chromatin fragments and occurred in a time-dependent and concentration-dependent manner. Results indicate that chromatin induces both neo-synthesis of soluble CEACAM8 and release of soluble CEACAM8 through degranulation. In addition, we report the presence of soluble CEACAM8 at high concentration in the synovial fluid of RA patients. Thus, we describe here a novel mechanism by which a natural DAMP, with inflammatory properties in SLE and RA, induces soluble CEACAM8 secretion by activated PMN with potential immunoregulatory consequences on other immune cells, including PMN.

## Introduction

Systemic lupus erythematosus (SLE) and rheumatoid arthritis (RA) are two inflammatory autoimmune and rheumatic diseases of unknown etiology and triggered by a combination of genetic and environmental factors as well as immune dysregulation. In addition to sepsis and cancers, extracellular chromatin is present in SLE and RA. In SLE, chromatin and especially mono-nucleosomes (the fundamental DNA packing unit, a complex of 180 base pairs of DNA and one copy of histone H1 and two copies of histones H2A, H2B, H3, and H4) are detected in the circulation of patients (1) as a result of both increased apoptosis and decreased clearance of apoptotic cells and extracellular chromatin. Chromatin represents a major autoantigen in SLE. IgG3 anti-nucleosome autoantibodies are associated with active disease (2). In RA patients, cell-free chromatin is detected in the synovial fluid of inflamed joints (3) and deposits in affected joints where they form immune complexes (4). In these patients, chromatin might be released by polymorphonuclear neutrophils (PMN) recruited into inflamed tissues and dying after activation, or part of it might derive from neutrophil extracellular traps (NET) released (NETosis) upon activation (5). We have shown that extracellular chromatin activates several innate immune cells, may behave as a danger-associated molecular pattern (DAMP) and might be pathogenic in RA and SLE. Indeed, chromatin triggers activation of dendritic cells from healthy donors (HD) and SLE patients (6). Moreover, it activates PMN from HD, SLE, and RA patients (7) in a Toll-like receptor (TLR) 9-independent manner (8), leading to secretion of pro-inflammatory cytokines as well as interferon (IFN)-α, a key cytokine in SLE, and induction of NET (9). In the latter studies, we also observed that once recognized, chromatin is endocytosed by PMN.

Importantly, PMN are activated in RA and SLE patients. Originally described as short lived cells, they can actually survive more than five days *in vivo* in humans (10). They probably participate to the pathogenesis of these inflammatory diseases as they are described as typical pro-inflammatory cells and are able to interact with the pro-inflammatory Th17 lymphocytes (11). In RA patients, PMN differentiate into dendritic-like cells (12) and express RANK-L (13), suggesting a role in osteoclastogenesis and bone destruction. Surprisingly, PMN remain relatively poorly studied in this context. Recent data suggest that PMN also exert regulatory (14) or even anti-inflammatory functions (15) and can behave as B lymphocyte-helper cells (16), indicating that a tight regulation is required *in vivo*. Particularly, the triggers involved, and especially the disease-specific or -associated ones, have to be better characterized. Likewise, the cross-talk between PMN and other cell types, especially with both innate and adaptive immune cells, have to be examined.

PMN express plasma membrane carcinoembryonic antigen-related cell adhesion molecule (CEACAM) 8 or CD66b, a glycosylphosphatidylinositol (GPI)-anchored glycoprotein which is solely expressed by granulocytes (17), of which 95% are PMN. CD66b is thus specific to granulocytes and is used as a granulocyte and even a PMN marker. CEACAM8 is stored in specific (secondary) granules (18). Due to its increased expression in stimulated PMN, it is also an activation marker as sign of rapid degranulation. In addition, upon stimulation PMN secrete a soluble variant of CEACAM8 (19). Particularly, release of soluble CEACAM8 reflects degranulation of secondary granules. Soluble CEACAM8 has immunoregulatory functions. Indeed, soluble CEACAM8 has chemotactic activities for lymphocytes (20) and is known to bind to plasma membrane CEACAM1 (CD66a) (21). The latter is expressed on epithelia, endothelial cells, and various leukocytes subtypes as two major splice variants, CEACAM1-S, and CEACAM1-L (22). Only the L-variant contains immunoreceptor tyrosine-based inhibitory motifs (ITIM) in its cytoplasmic domain. Binding of soluble CEACAM8 to plasma membrane CEACAM1 triggers different functions like a co-stimulatory activity on B lymphocytes (20) or inhibition of TLR2 response in lung epithelial cells (23), and thus an anti-inflammatory signal through binding to plasma membrane CEACAM1 (24). As CEACAM are cell-cell communication molecules, soluble CEACAM8 may only be detected on other cells as a result of binding with other membrane CEACAM. Thus, the combination of stimuli encountered *in vivo* may dictate soluble CEACAM8 immunoregulatory effects. However, the natural stimuli inducing soluble CEACAM8 secretion need to be characterized. Therefore, we have investigated whether the cell-free DAMP chromatin can trigger soluble CEACAM8 secretion upon PMN activation. Moreover, we have tested whether soluble CEACAM8 is abnormally secreted in RA patients.

## Methods

### Human Samples

EDTA-blood from random, healthy individuals (blood bank of Bobigny, contract 13/A/107, France), and RA patients (Rheumatology Department, Avicenne Hospital, Bobigny, France) was used. RA patients fulfilled the American College of Rheumatology-European League Against Rheumatism 2010 criteria. We focused on RA patients who were not treated with biologic therapy. All RA patients had a history of positivity for anti-citrullinated protein antibodies (ACPA). Blood was used to prepare leukocytes and plasma. Fresh cell-free synovial fluids were also collected from RA patients as well as patients with gout or osteoarthritis. Informed consents were collected. Experiments were approved by the local ethics committee CPP Paris Ile de France (NI-2016-11-01).

### Chromatin Purification

Chromatin and nucleosomes were prepared under sterile conditions from calf thymus as previously described (6, 25). Briefly, nuclei were isolated and then digested by micrococcal nuclease (Sigma-Aldrich). The reaction was stopped by EDTA and centrifuged. The pellet was harvested and nuclei were lyzed. After centrifugation, the supernatant containing chromatin was collected. This fraction is composed of chromatin fragments of different sizes, including high molecular weight complexes. When used in cell culture, the lysis buffer served as a negative control. In other experiments, mono-nucleosomes were used. In that case, chromatin was further purified by ultracentrifugation on 5–29% sucrose gradients. As a negative control in cell culture, the purification buffer was used, i.e., an empty sucrose gradient loaded with lysis buffer only. All chromatin fractions were analyzed by agarose gel electrophoresis (1.5%) and SDS-PAGE (18%). Of note, free self DNA is not strongly immunogenic and histones are 100% conserved in human and calf.

### Cell Isolation and Culture

Polymorphonuclear neutrophils and peripheral blood mononuclear cells (PBMC) were freshly isolated by dextran sedimentation (Axis Shield) from peripheral blood as previously described (7). Contaminating red blood cells were lysed using cold ACK hypotonic buffer (NH_4_Cl, KHCO_3_, and EDTA). PMN purity was estimated by flow cytometry (Supplementary Figures 1A,B).

PMN (defined as CD66b^+^, CD11b^+^, CD3^−^, CD19^−^, CD56^−^ cells, purity >95% of living cells) were cultured (10^6^ cells/ml) in RPMI 1640 medium (Gibco) supplemented with 10% heat-inactivated fetal calf serum (FCS, biowest) in the presence/absence of 5 ng/ml phorbol myristate acetate (PMA), 5 ng/ml lipopolysaccharides (LPS, from *S. typhimurium*, a TLR4 agonist, all from Sigma-Aldrich), 2 μM synthetic oligonucleotide containing unmethylated CpG motifs (a TLR9 agonist, InvivoGen) or purified chromatin or its purification buffer as a control. In some cases, activation studies were performed with 25 μg/ml polymyxin B (an inhibitor of LPS, Sigma-Aldrich) or after pre-incubation at 56°C for 1 h to induce heat shock. Cell activation was estimated after 0.5–14 h by flow cytometry and by measuring cytokine secretion in cell culture supernatants by ELISA. Secretion of soluble CEACAM1, soluble CEACAM6, and soluble CEACAM8 was determined by ELISA.

### Flow Cytometry

Purity and phenotype of PMN were determined by staining with monoclonal antibodies (mAb) specific for CD66b (FITC-conjugated, clone G10F5) or CD11b (PE-conjugated, clone ICRF44), CD3 (PerCP-conjugated, clone UCHT-1), CD19 (APC-conjugated, clone LT19), CD56 (PE-conjugated, clone B159), or the corresponding isotype control, at 4°C in staining buffer (PBS containing 5% heat-inactivated FCS, 100 μg/ml human γ-globulin (Calbiochem), 0.02% sodium azide), and according to classical protocols. Cell viability was estimated by propidium iodide (PI) staining. To analyze PMN activation, plasma membrane CD11b expression levels were estimated on CD66b-positive cells after staining with mAb specific for CD66b and CD11b. All antibodies were purchased from BD Biosciences (except anti-CD3 and anti-CD19, ImmunoTools). PMN activation was confirmed by measuring oxidative burst and the phagocytic activity after incubation with dichlorofluorescin diacetate (DCFDA, Sigma, 25 μM) or phycoerythrin-labeled polystyrene microspheres (1 μm in diameter, Fluoresbrite Plain Microspheres PCRed, Polysciences, 5 ×10^6^ microbeads for 2 ×10^5^ PMN), respectively, for 2 h at 37°C and then fixation in 1% paraformaldehyde. DCFDA becomes green fluorescent when oxidized in dichlorofluorescein (DCF). In some cases, fresh untouched cells were directly stained in whole blood after red blood cell lysis to compare PMN and PBMC using different gates. Cells were analyzed on a four-color FACSCalibur apparatus (Becton Dickinson). Data were evaluated with CellQuest Pro software (Becton Dickinson). Plasma membrane CD66b and CD11b expression levels (mean fluorescence intensity, MFI) are depicted as mean ± standard deviation (SD) of triplicates.

### ELISA

Detection of secreted soluble CEACAM1, soluble CEACAM6, and soluble CEACAM8 was performed using self-established sandwich ELISA as reported previously (23). Briefly, plates (Costar) were coated with polyclonal rabbit antibody against human CEA (DAKO, 5 μg/ml). Remaining binding sites were blocked with PBS-BSA (3%, Sigma). Plates were then incubated with culture supernatants. Standards were prepared using CEACAM1-Fc, CEACAM6-Fc, and CEACAM8-Fc proteins. Bound CEACAM were detected with 10 μg/ml anti-CEACAM1 (clone 18/20), anti-CEACAM6 (clone 1H7-4B), or anti-CEACAM8 (clone 6/40/c) mAb. Then, peroxidase-conjugated AffiniPure goat anti-mouse IgG antibody (Jackson ImmunoResearch, diluted 1:5000) was incubated. Enzyme reaction was visualized using TMB (Sigma) as substrate and stopped with H_2_SO_4_. Absorbance was measured at 450 nm.

Interleukin (IL)-8 and IL-10 secretion by human PMN was quantified by sandwich ELISA using OptEIA set (BD Biosciences) or mAb pair and streptavidin-peroxidase conjugate (eBioscience) and according to the manufacturer's instructions.

Soluble CEACAM and cytokine concentrations in cell culture supernatants are depicted as mean ± SD of triplicates. Soluble CEACAM8 concentrations in plasmas and synovial fluids are depicted as mean ± standard error of the mean (SEM).

### Statistical Analysis

For cell cultures, representative experiments are presented and the numbers of independent experiments using different donors and different chromatin preparations are indicated. Levels of plasma membrane CD66b/CD11b expression or soluble CEACAM/IL-8 secretion are depicted as mean and SD of triplicates of the representative culture. In addition, significance of differences between chromatin/nucleosomes and the purification buffer has been tested in individual experiments using a two-tailed unpaired *t*-test with or without Welch's correction. Correlations between soluble CEACAM concentrations and expression levels of plasma membrane CD66b and CD11b were assessed by using two-tailed Spearman tests. Soluble CEACAM8 concentrations in plasma from HD and RA patients were compared using a two-tailed Mann-Whitney test. Soluble CEACAM8 concentrations in all RA plasma and RA synovial fluids were compared using a two-tailed Mann-Whitney test. Soluble CEACAM8 concentrations in RA patients for whom plasma and synovial fluid were collected in parallel were compared using a two-tailed Wilcoxon signed rank test. Percentages of HD and RA patients with high concentrations of circulating soluble CEACAM8 were compared using Fisher's test. Data were analyzed using GraphPad Prism software (*p* < 0.05 was considered significant).

## Results

### Cell-Free Large Extracellular Chromatin Fragments Trigger Secretion of Soluble CEACAM6 and CEACAM8 by PMN

**Figure 1:**
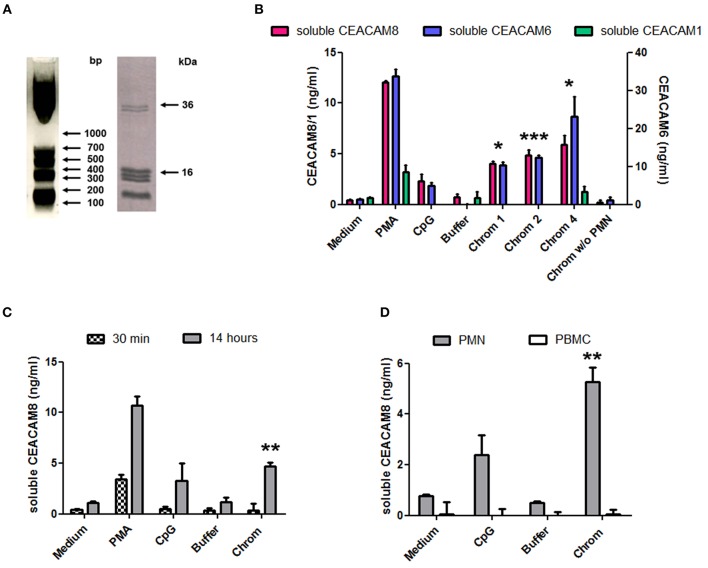
Extracellular chromatin fragments of different sizes trigger the release of soluble CEACAM8 by activated PMN. **(A)** Chromatin was obtained after nuclease digestion and lysis of nuclei and was then analyzed by 1.5% agarose gel electrophoresis (left) and 18% SDS-PAGE (right). Molecular weight markers are indicated. bp, base pairs. The doublet at ~35 kDa represents histone H1. **(B)** Freshly isolated human PMN were cultured for 14 h at 10^6^ cells/ml with different stimuli and the release of soluble CEACAM1, CEACAM6, and CEACAM8 was estimated in the supernatants by ELISA. PMA, phorbol myristate acetate; CpG, oligonucleotide containing unmethylated CpG motifs (TLR9 agonist); buffer, nuclei lysis buffer; Chrom, chromatin (1, 2, 4 indicate concentrations in μg/ml). As a control, chromatin was incubated without (w/o) PMN. **(C)** PMN were activated as in **(B)** for 14 h or 30 min and secretion of soluble CEACAM8 was estimated (4 μg/ml chromatin was used). **(D)** PMN and autologous PBMC were cultured and activated (4 μg/ml chromatin) in parallel for 14 h and then secretion of soluble CEACAM8 was determined. Shown is one representative experiment of seven independent experiments using different donors and different chromatin preparations. Mean and SD of triplicates are shown. *p < 0.05; **p < 0.01; ***p < 0.001 for soluble CEACAM8/6 concentrations after culture with chromatin vs. the purification buffer.

**Figure 2:**
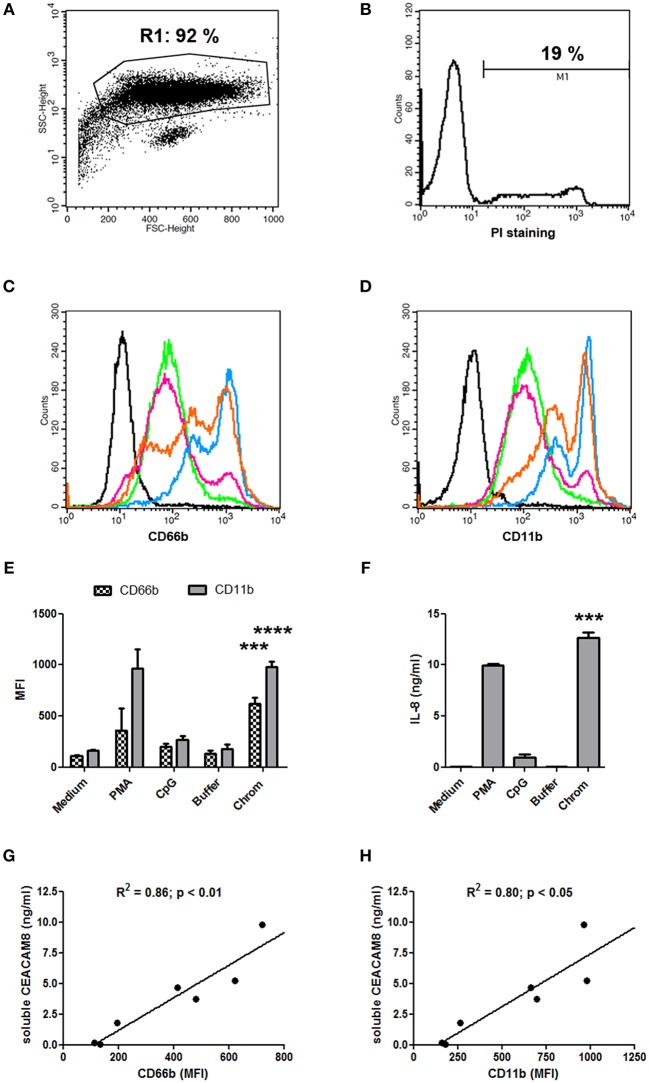
Chromatin-induced soluble CEACAM8 secretion is associated with PMN activation. **(A,B)** Classical PMN shape **(A)** after 14 h of cell culture in medium only (non-activated PMN) and percentage of dead cells **(B)**. PMN were analyzed by flow cytometry after staining with propidium iodide (PI). Size (FSC) and granularity (SSC) are depicted. Numbers represent percentages of gated cells and dead (PI-positive) PMN. **(C–F)** After 14 h, cell activation was estimated by flow cytometry **(C–E)** or ELISA **(F)** with PMN from Figure 1C. **(C,D)** Representative plasma membrane CD66b (CEACAM8) **(C)** and plasma membrane CD11b **(D)** expressions after chromatin activation are represented. Black histogram, PMN in medium stained with isotype control. All other histograms represent PMN stained with CD66b- or CD11b-specific mAb; green, PMN in medium; pink, PMN with chromatin purification buffer; orange, PMN with 1 μg/ml chromatin; blue, PMN with 4 μg/ml chromatin. **(E)** CD66b (CEACAM8) and CD11b expression levels for all stimuli are summarized (chromatin, 4 μg/ml). MFI, mean fluorescence intensity. **(F)** IL-8 secretion was quantified (chromatin, 4 μg/ml). **(G,H)** Correlations between concentrations of secreted soluble CEACAM8 and levels of plasma membrane CD66b (CEACAM8) **(G)** and CD11b **(H)** expression by activated PMN were determined using two-tailed Spearman tests. Shown is one representative experiment of seven independent experiments using different donors and different chromatin preparations. Mean and SD of triplicates are shown. ***p < 0.001; ****p < 0.0001 for PMN cultured with chromatin vs. the purification buffer.

The capacity of extracellular chromatin to trigger secretion of soluble CEACAM8 (CD66b) by PMN was investigated. We focused on chromatin and not free histones or DNA for several reasons. Free extracellular histones and DNA are usually not observed at high concentrations in RA and SLE patients. Most of extracellular histones or DNA is rather detected in chromatin, i.e., DNA complexed with histones, and eventually additional associated proteins. Moreover, we have previously reported that, in contrast to chromatin, histones do not activate PMN (7) or dendritic cells (6). On the other hand, it should also be noted that free mammalian DNA is usually poorly stimulatory. Free DNA can only efficiently trigger activation of innate immune cells when it is forced to enter cells or to reach endosomes or when it is present in immune complexes or when it is opsonized e.g., by histones, like in chromatin. Likewise, we have previously shown that extracellular chromatin triggers PMN activation but not free DNA, even DNA purified from chromatin (7). Thus, we first tested chromatin fragments of different sizes using nuclease-digested and lyzed nuclei, without further purification by ultracentrifugation. These preparations contain a mixture of nucleosomal oligomers and larger nucleosomal complexes, as evidenced by DNA size (with individual bands and not a smear), and the presence of the five histones (Figure 1A). Extracellular chromatin triggers the release of soluble CEACAM8 by human PMN *in vitro* (Figure 1B) in a concentration-dependent manner, in contrast to the purification buffer (which is the true negative control). PMA and the TLR9 agonist also induce soluble CEACAM8 as previously reported (23). As a control, we verified that no signal was detected in the absence of PMN, excluding that CEACAM-specific antibodies cross-react with chromatin (Figure 1B). Interestingly, chromatin also induces soluble CEACAM6 (CD66c) release, but not soluble CEACAM1 (Figure 1B). Chromatin only induces strong soluble CEACAM8 secretion after 14 h and not after 30 min, in contrast to PMA which is known to induce degranulation, and thus vesicle release, within minutes (Figure 1C). These two time points were used to compare the fast release of pre-stored soluble CEACAM8 from intracellular granules (which takes just minutes) to the secretion of soluble CEACAM8 after several hours of stimulation, allowing neo-synthesis (thus transcription and translation) as previously described (23). Actually, secretion of neo-synthesized soluble CEACAM8 is already detectable after 6 h (Supplementary Figure 2) but differences between non-stimulated and stimulated PMN are amplified after 14 h, because soluble CEACAM8 is accumulated in supernatants over time after stimulation without increasing spontaneous secretion in non-stimulated PMN. Induction of some PMN functions requires priming, i.e., pre-activation e.g., by granulocyte-macrophage colony-stimulating factor (GM-CSF) before stimulation. Importantly, we observed that chromatin directly triggers soluble CEACAM8 secretion without requirement of PMN priming as no cytokine was used. GM-CSF pre-sensitizes PMN but did not trigger soluble CEACAM8 release (data not shown). Likewise, chromatin does not require immune complex formation to release soluble CEACAM8 as no autoantibody was used. We also confirmed that soluble CEACAM8 is specifically secreted by activated PMN, and not by autologous PBMC (Figure 1D). Furthermore, we confirm in our system published data showing that CD66b (CEACAM8) is specifically expressed by PMN in whole blood (Supplementary Figures 1C–E). Chromatin-induced soluble CEACAM8 secretion was associated with PMN activation, as shown by plasma membrane CD66b (CEACAM8) and CD11b up-regulation (Figures 2A–E) and IL-8 secretion (Figure 2F). Using optimized cell culture conditions, 92% of cells still have a typical PMN shape after 14 h (Figure 2A) with only 19% of dead cells (Figure 2B), as estimated by flow cytometry. It should be noted that PMN viability is even higher upon activation (Supplementary Figure 3), where cell activation (estimated by CD11b up-regulation) is inversely associated with cell death. Other groups have actually already reported similar culture conditions (26). Extracellular chromatin up-regulates both plasma membrane CD66b (CEACAM8) (Figures 2C,E) and CD11b (Figures 2D,E) in a concentration-dependent manner. At 14 h post PMN activation, levels of soluble CEACAM8 and plasma membrane CEACAM8 (Figure 2G) as well as plasma membrane CD11b (Figure 2H) were strongly positively correlated. Next, to exclude release of soluble CEACAM8 by dying PMN, cells were treated at 56°C to induce heat shock and subsequent death. No clear secretion of soluble CEACAM8 was observed when PMN were treated either for 1 h at 56°C, or when they were pre-treated at 56°C and then cultured for 14 h in medium (Supplementary Figure 4). On the contrary, secretion of soluble CEACAM8 was even reduced (−24%) when PMN were pre-treated at 56°C in the presence of PMA and then cultured for additional 14 h. All these controls prove that secretion of soluble CEACAM8 is not a consequence of PMN stress and death. Moreover, we have previously shown that chromatin is not toxic for PMN (7).

### Mono-Nucleosomes Activate PMN to Secrete Soluble CEACAM8

**Figure 3:**
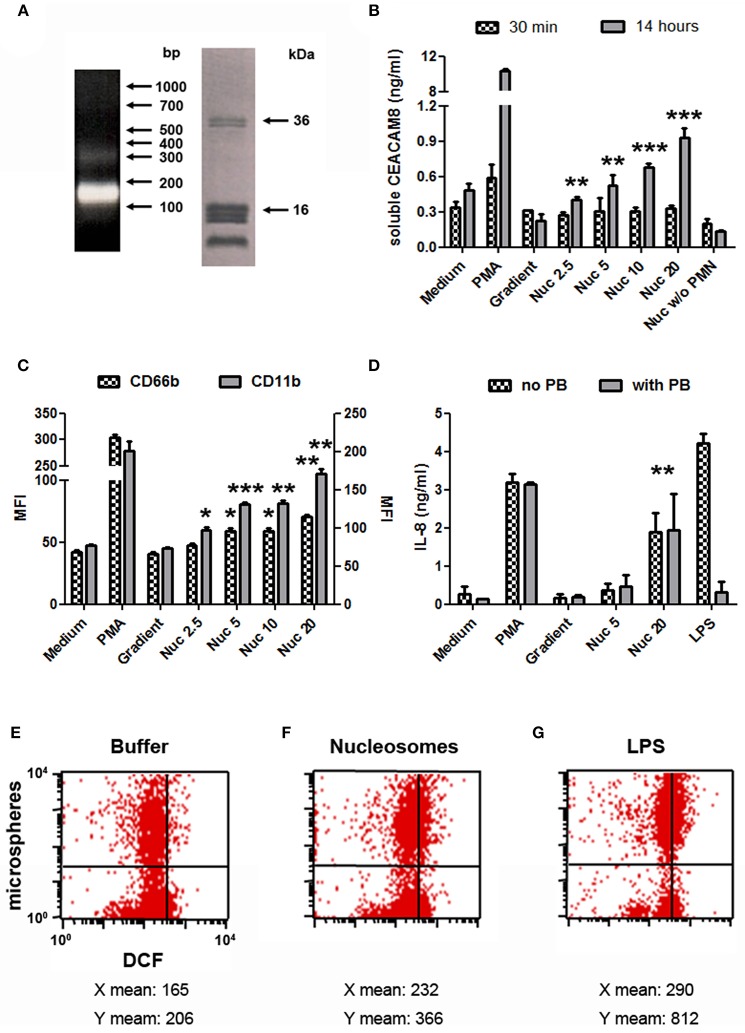
Mono-nucleosome-induced PMN activation leads to secretion of soluble CEACAM8. **(A)** Chromatin was further purified by ultracentrifugation on 5–29% sucrose gradients to get mono-nucleosomes. Purified nucleosomes were analyzed by 1.5% agarose gel electrophoresis (left) and 18% SDS-PAGE (right). Molecular weight markers are indicated. bp, base pairs. The doublet at ~35 kDa represents histone H1. **(B)** Freshly isolated human PMN were cultured for 30 min or 14 h with different stimuli and the release of soluble CEACAM8 was estimated in the supernatants by ELISA. PMA, phorbol myristate acetate; Gradient, empty sucrose gradient loaded with nuclei lysis buffer instead of chromatin; Nuc, purified mono-nucleosomes (2.5, 5, 10, 20 indicate concentrations in μg/ml). As a control, nucleosomes were incubated without (w/o) PMN. **(C,D)** PMN were activated as in **(B)** for 14 h and cell activation was estimated by flow cytometry **(C)** or ELISA **(D)**. Plasma membrane CD66b (CEACAM8) and CD11b expression and IL-8 secretion were determined. MFI, mean fluorescence intensity. In **(D)**, PMN were cultured with or without polymyxin B (PB, a LPS antagonist). Shown is one representative experiment of three independent experiments using different donors and different purifications of nucleosomes. Mean and SD of triplicates are shown. **(E–G)** Chromatin-induced PMN activation leads to increased phagocytic activity and oxidative burst. Freshly isolated human PMN were cultured for 2 h in medium supplemented with the chromatin purification buffer **(E)** or stimulated with 20 μg/ml purified mono-nucleosomes **(F)** or 5 ng/ml LPS **(G)**, in the presence of dichlorofluorescin diacetate (which is oxidized in dichlorofluorescein (DCF), x axis) and phycoerythrin-conjugated microspheres (y axis) to measure oxidative burst and phagocytosis, respectively, by flow cytometry. Shown is one representative experiment of three independent experiments using different donors. Mean fluorescence intensities for both axes are depicted below each corresponding dot-plot. LPS, lipopolysaccharides. *p < 0.05; **p < 0.01; ***p < 0.001 for PMN cultured with nucleosomes vs. the purification buffer.

We next focused on purified mono-nucleosomes to refine activation-induced soluble CEACAM8 release. Mono-nucleosomes are indeed a main nucleosomal complex observed in the circulation of SLE patients and are deposited in joints of RA patients. We only analyzed soluble CEACAM8 because this CEACAM is specific to granulocytes. After digestion/lysis of nuclei, chromatin was further purified on sucrose gradients by ultracentrifugation. Fractions containing mono-nucleosomes were collected. These preparations essentially contain mono-nucleosomes (180 base pairs of DNA and the five histones, Figure 3A). Using purified nucleosomes, we confirmed induction of soluble CEACAM8 secretion by PMN (Figure 3B). Nucleosomes induced soluble CEACAM8 in a dose-dependent manner, as compared to the empty gradient (the purification buffer), which is the true negative control. No signal was observed in the absence of PMN, confirming the specificity of the CEACAM8 signal. Anew, soluble CEACAM8 secretion was detected after 14 h, but not after 30 min. Nucleosome-induced soluble CEACAM8 secretion was also associated with PMN activation, as shown by plasma membrane CD66b (CEACAM8) and CD11b up-regulation (Figure 3C) and IL-8 secretion (Figure 3D). We also excluded that nucleosome-induced PMN activation was due to endotoxin contamination as it was not inhibited by polymyxin B, in contrast to LPS (Figure 3D). PMN activation in response to extracellular mono-nucleosomes was also confirmed in some donors by showing both increased oxidative burst and increased phagocytic activity (Figures 3E–G). On the contrary, NETosis was not observed in response to chromatin (data not shown) and thus was not associated with soluble CEACAM8 release. We have recently shown that NET activate macrophages and PMN, especially in RA (27). However, different and optimized experimental settings were used for NET induction. Likewise, IL-10 secretion by chromatin-activated PMN was not observed (data not shown), suggesting that PMN with an immuno-modulatory activity were not triggered.

### Concentrations of Soluble CEACAM8 Are Elevated in Inflamed Joints of RA Patients

**Figure 4:**
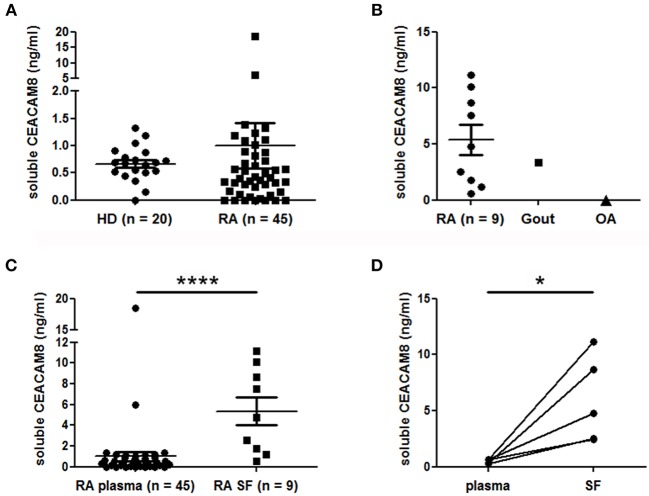
Soluble CEACAM8 is enriched in inflamed tissue of patients suffering from rheumatoid arthritis. **(A)** Circulating soluble CEACAM8 concentrations were estimated by ELISA in the plasma of healthy donors (HD) and rheumatoid arthritis (RA) patients. **(B)** Concentrations of soluble CEACAM8 were determined in the synovial fluid of nine RA patients, one gout patient and one osteoarthritis (OA) patient. **(C)** Comparison of soluble CEACAM8 concentrations in all RA plasmas and all RA synovial fluids tested. **(D)** Comparison of soluble CEACAM8 concentrations in the plasma and the synovial fluid of five RA patients. Mean and SEM are presented. *p < 0.05; ****p < 0.0001.

To support a potential role of soluble CEACAM8 in inflammatory autoimmune diseases, we measured and compared concentrations of soluble CEACAM8 in the plasma of HD and patients with RA, a disease with pathogenic involvement of PMN. Low levels of soluble CEACAM8 were detected in both HD (mean = 0.67 ng/ml) and RA patients (mean = 0.99 ng/ml) with no significant statistical difference (Figure 4A). Only two RA patients (4.4 vs. 0% in HD, not significant) showed high soluble CEACAM8 concentrations that could not be explained by clinical data. To determine whether soluble CEACAM8 is produced locally in inflamed tissues rather than systemically, soluble CEACAM8 was measured in synovial fluid. Interestingly, we show for the first time that soluble CEACAM8 is present at high concentration in the synovial fluid of inflamed joints from RA patients (Figure 4B, mean = 5.4 ng/ml), suggesting that soluble CEACAM8 is enriched in affected tissues. Interestingly, elevated soluble CEACAM8 concentration was also observed in the synovial fluid of one patient with gout (an inflammatory disease with strong PMN influx), whereas no soluble CEACAM8 was detected in the synovial fluid of one patient with osteoarthritis (a non-inflammatory disease with low PMN influx). When all RA samples were compared, soluble CEACAM8 concentrations were significantly higher in the synovial fluid than in the plasma (Figure 4C, *p* < 0.0001). Particularly, in the five RA patients for whom we obtained simultaneously plasma and synovial fluid, concentrations of soluble CEACAM8 were significantly increased in all the synovial fluids when compared to plasmas (Figure 4D, *p* < 0.05).

## Discussion

We demonstrate here for the first time that extracellular chromatin induces secretion of soluble CEACAM8, a molecule with immuno-modulatory functions, after activation of human PMN. Concentrations of soluble CEACAM8 measured *in vitro* are similar to concentrations measured in synovial fluids and are therefore physiologic concentrations. Thanks to its diverse activities and via its binding to plasma membrane CEACAM1, which is expressed on different cell types including PMN (28), soluble CEACAM8 is involved in the control of immune responses and PMN communication, not only in PMN-PMN communication but also in the cross-talk with other innate immune cells and even in PMN-adaptive immunity cross-talk. Because, extracellular chromatin is a major DAMP detected in RA and SLE patients and triggering sterile inflammation, this mechanism may participate to the pathogenesis of these inflammatory diseases.

Our kinetics study suggests that chromatin directly triggers both neo-synthesis and subsequent secretion of soluble CEACAM8 rather than the rapid release of CEACAM8 pre-stored in granules, as observed with PMA. Chromatin also triggers plasma membrane-bound CEACAM8 (CD66b) up-regulation. As expected, soluble CEACAM8 was only secreted by activated PMN, and not by autologous PBMC, as CEACAM8 (CD66b) is a granulocyte marker. Because soluble CEACAM8 release was positively correlated with plasma membrane-anchored CEACAM8 and CD11b expression, this process is associated with degranulation and PMN activation. PMN activation was confirmed by increased IL-8 secretion, phagocytic activity as well as oxidative burst, whereas IL-10 secretion and NETosis were not observed.

Importantly, we also demonstrate for the first time that soluble CEACAM8 is present at high concentrations in RA synovial fluids, whereas its concentration was low in the plasma and comparable to that of HD. This suggests that soluble CEACAM8 is enriched in inflamed RA joints. These synovial fluids are also known to be enriched in PMN and extracellular chromatin. Soluble CEACAM8 might be released locally after activation of recruited PMN and may amplify the inflammatory process. Of note, membrane-bound CEACAM8 (CD66b) is up-regulated on PMN isolated from RA synovial fluid (29).

Further studies will be necessary to determine how chromatin triggers soluble CEACAM8 release. We have already reported that free mammalian DNA isolated from purified nucleosomes and purified histones do not activate PMN (7), suggesting that the nucleosomal structure is important to trigger activation. In addition, we have previously shown that TLR2/4, endosomal acidification, and TLR9 [including the cell surface TLR9 we described on PMN (30)] are not required for nucleosome-induced PMN activation (7, 8). Nevertheless, other intracellular DNA sensors might be involved (31), like AIM2 (32), DAI (33), or STING (34), the latter being up-regulated by extracellular chromatin (9) and potentially involved in lupus pathogenesis for example.

In conclusion, extracellular chromatin triggers a strong secretion of soluble CEACAM8, which may lead to an over-reaction of the immune system by interaction with a broad range of immune cells. Studies are currently performed to determine whether NET also trigger soluble CEACAM8 secretion. Indeed, we have recently reported that NET, and especially RA NET, are pro-inflammatory and activate PMN and macrophages (27), supporting the pathogenic role of PMN in RA. Finally, we are measuring concentrations of soluble CEACAM8 in samples from patients suffering from other autoimmune diseases.

## Ethics Statement

EDTA-blood from random, healthy individuals (blood bank of Bobigny, contract 13/A/107, France), and RA patients (Rheumatology Department, Avicenne Hospital, Bobigny, France) was used. Informed consents were collected. Experiments were approved by the local ethics committee CPP Paris Ile de France (NI-2016-11-01).

## Author Contributions

All authors were involved in drafting the manuscript. PD designed the research, performed part of the experiments, analyzed, and interpreted data, and wrote the manuscript. MR performed the experiments, analyzed, and interpreted data. JM performed part of the experiments and analyzed data. LS selected patients and analyzed data. BS contributed reagents, analyzed, and interpreted data.

### Conflict of Interest Statement

The authors declare that the research was conducted in the absence of any commercial or financial relationships that could be construed as a potential conflict of interest.
