# Supplementary material for: Extracellular Chromatin Triggers Release of Soluble CEACAM8 Upon Activation of Neutrophils
Source: Front Immunol. 2019 Jun 14;10:1346. doi: 10.3389/fimmu.2019.01346 (PMC6587075; doi:10.3389/fimmu.2019.01346)
Supplement: Supplementary file 1 [file Presentation_1.pdf]

Supplementary Material

Supplementary Figures

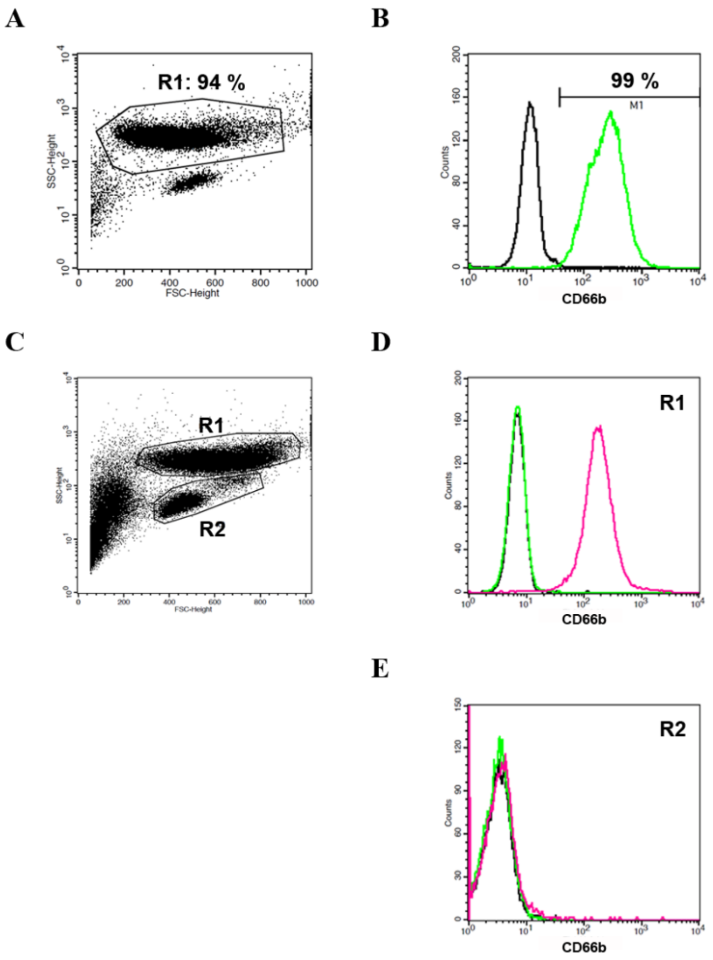

Supplementary Figure 1. Purity of isolated human PMN and specificity of the CD66b staining. (A, B) Freshly isolated PMN were stained ex vivo for membrane CD66b and analyzed by flow cytometry. **(A)** Dot-plot representing size and granularity. Gated PMN represent 94 % of all events. **(B)** Histograms depicting CD66b expression. Black histogram, PMN stained with isotype control; green histogram, PMN stained with the CD66b-specific mAb. 99 % of cells were positive for the PMN marker CD66b. **(C-E)** Untouched total leukocytes were stained ex vivo in whole blood. **(C)** Dot-plot representing size and granularity. Gates representing PMN (R1) and PBMC (R2) are depicted. Gated PMN from R1 **(D)** and gated PBMC from R2 **(E)** were analyzed for plasma membrane CD66b expression. Black histograms, unstained cells; green histograms, cells stained with isotype control; pink histograms, cells stained with the CD66b-specific mAb. Shown is one representative experiment of at least 20 independent experiments using different donors.

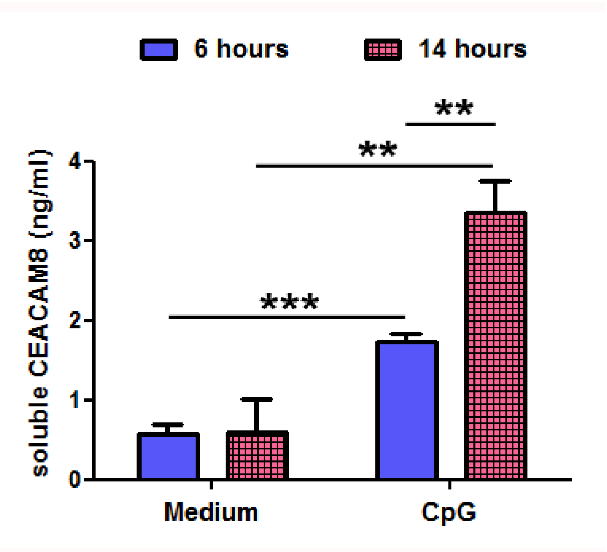

Supplementary Figure 2. Secretion of neo-synthetized soluble CEACAM8 by human PMN is detectable 6 hours after stimulation. Freshly isolated human PMN were cultured for 6 or 14 hours in medium only or stimulated by a TLR9 agonist (CpG). Cell culture supernatants were then collected and concentrations of secreted soluble CEACAM8 were determined by ELISA. Shown is one representative experiment of 3 independent experiments using different donors. Mean and SD of triplicates are shown. CpG, oligonucleotide containing unmethylated CpG motifs. **, $p < 0.01$; ***, $p < 0.001$.

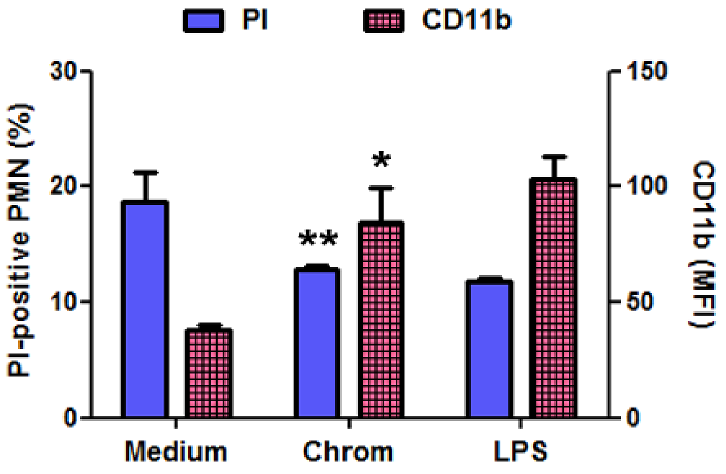

Supplementary Figure 3. PMN activation increases survival. Freshly isolated human PMN were cultured for 14 hours in medium only or stimulated with extracellular chromatin or LPS. Cell death and cell activation were then estimated by flow cytometry after staining with propidium iodide or CD11b-specific mAb, respectively. Percentages of dead PMN (PI-positive, left axis) and CD11b expression levels (right axis) are depicted. Chrom, 4 µg/ml purified chromatin; LPS, 5 ng/ml lipopolysaccharides. Shown is one representative experiment of at least 10 independent experiments using different donors. Mean and SD of triplicates are shown. PI, propidium iodide; MFI, mean fluorescence intensity. *, $p < 0.05$; **, $p < 0.01$ for PMN cultured with chromatin versus medium.

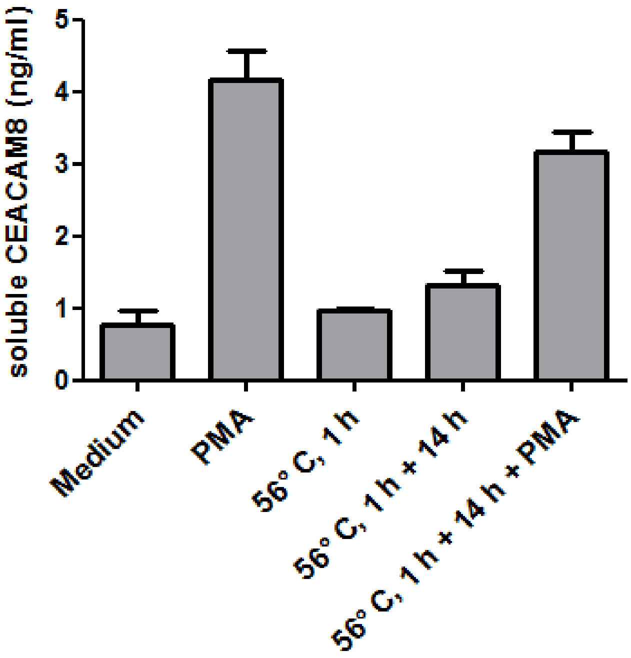

Supplementary Figure 4. Soluble CEACAM8 release is not a consequence of PMN stress and death. Freshly isolated human PMN were either cultured for 14 hours in medium only or stimulated by PMA (which induces both up-regulation of plasma membrane anchored CEACAM8 and soluble CEACAM8), or treated at 56° C for one hour (h), or pre-treated at 56° C for one hour and then cultured for 14 hours in medium, or pre-treated at 56° C for one hour in the presence of PMA and then cultured for 14 hours. Cell culture supernatants were then collected and concentrations of secreted soluble CEACAM8 were determined by ELISA. Shown is one representative experiment of 3 independent experiments using different donors. Mean and SD of triplicates are shown.
